# Cancer drug resistance: rationale for drug delivery systems and targeted inhibition of HSP90 family proteins

**DOI:** 10.20517/cdr.2019.26

**Published:** 2019-09-19

**Authors:** Clélia Mathieu, Samir Messaoudi, Elias Fattal, Juliette Vergnaud-Gauduchon

**Affiliations:** ^1^Institut Galien Paris-Sud, CNRS, UMR 8612, Université Paris-Sud/Paris-Saclay, Châtenay-Malabry 92296, France.; ^2^BioCIS, CNRS, UMR 8076, Université Paris-Sud/Paris-Saclay, Châtenay-Malabry 92296, France.

**Keywords:** Heat-shock proteins, cancer, resistance, nanoparticles

## Abstract

Nanocarriers have been developed in order to protect drugs or to improve drugs efficiency by reaching the damaged tissue and avoiding systemic and local toxicity. By using HSP90 inhibitors, some cancer drug resistances have been overcome and the loading into nanocarriers of such drugs has shown an increase of their activities. This review will present some advantages of HSP90 inhibitors to treat resistant tumors; especially those targeting the mitochondrial protein TRAP1. We will also focus on the targeting of the primary tumors, cancer stem cells and metastatic cells.

## Introduction

According to the World Health Organization, 9.6 million of death are imputed to cancers in 2018. Despite lots of progress in cancer therapy, there are still lots of relapses and resistant cancers. In spite of a large scope of targets for anti-cancer drugs, the use of inhibitors and drugs may lead to development of resistances. The study of the drug resistance mechanisms allowed the development of new drugs which are more efficient and thus is a promising scope of study to improve oncologic treatments. Some cancers developed resistance to multiple drugs, and it is primordial to find how to reverse this mechanism. Even if new strategies are promising a large panel of researches focus on the development of drug delivery systems. This review will combine the rationale to use nanocarriers to improve the efficiency of anticancer drugs with the role of Heat Shock Proteins (HSP) in the drug resistance and how inhibition of specific isoforms is a promising strategy to sensitize cancer cells to death.

## Drug delivery systems, the way to overcome cancer resistance to therapy

As drug resistance is not only due to genetic alterations, several hallmarks have been identified to represent mechanisms of resistance: overexpression of drug efflux pumps [P-glycoprotein (P-gp)], activation of pro-proliferative and/or prosurvival (antiapoptotic) pathways, metabolic changes which confer to heterogeneous cancer cell populations differential dependency to glucose and to biosynthetic blocks and finally modulation of tumor microenvironment (inflammatory, immune cells and fibroblasts)^[1,2]^. Some of these mechanisms affect cancer stem cells which have plasticity properties and which are responsible for the maintenance of tumors despite treatment and induce poor clinical outcome for patients^[3]^.

A major point which in part explains the low efficacy of conventional or targeted therapies to fight cancer cells is the compensatory mechanism that is used by these cells displaying redundancy between pathways. For example, even if one signaling molecule is inhibited by therapy, another one involved in a secondary pathway or being downstream of the first one activates a similar cellular program which confers proliferative properties^[1]^. Then, strategies have to focus on multiple pathways to increase chances to be effective with treatment. A family of proteins has been identified as a controller of signaling pathways: The Heat Shock Proteins. They are implicated in many physiological processes by preventing mis(un-)folding, denaturation of proteins. In cancer cells, these functions are associated with stabilized pro-tumoral protein like growth factor receptors, transcription factors, … The HSP family will be more deeply described in the last part of this review by focusing on HSP targeting. Nevertheless, two decades ago first evidences appeared in relation to the involvement of HSP in resistance mechanisms. As the HSPs are induced *in vitro* by cytotoxic drugs, it has been hypothesized that HSP would play an important role in the tumor cell tolerance. For example, in 1998, Vargas-Roig *et al.*^[4]^ published a breast cancer patient-based study showing a correlation between a high nuclear level of HSP70 in tumor cells and drug resistance. The combination of HSP27 and HSP70 correlated strongly with the disease-free survival of patients^[4]^. More recently, a review has summarized the importance of HSP in the chemoresistance of ovarian cancer^[5]^. It has been demonstrated that treatment of ovarian cancer cells induced HSP27, HSP70 and HSP90 as well as HSP60 and TNF receptor-associated protein 1 (TRAP-1) and the use of HSP inhibitors was shown to be active against the growth of resistant tumors. Nevertheless the authors of this review concluded that strategy using HSP inhibition has to be chosen patient by patient^[5]^. The transcription factor heat shock factor 1 (HSF-1) activates the transcription of HSP genes and has been associated to resistance mechanisms. Particularly, mammary tumorigenesis displays a link between HSF-1 and ERBB2 the Human epidermal growth factor receptor 2 (HER2). ERBB2 has been identified as a client protein of HSP90 machinery and in lapatinib-resistant ERBB2-positive breast cancer cells HSF1-mediated heat shock pathway is responsible for the adaptation of the receptor tyrosine kinase kinome of cancer cells. Interestingly these cells are sensitive to inhibition of HSF-1 and HSP90 confirming the therapeutic potential of the targeting HSP system^[6]^.

Besides the potential role of HSP inhibition in overcoming the multidrug resistances, some groups focused their research on the development of strategies involving combination therapies, inhibition of the drug efflux pumps, and of ABC cassettes^[7-9]^. Combination therapies are more and more developed as standard of care for resistant cancers: that includes RVD [Lenalidomide (immunomodulatory drug), Bortezomib (proteasome inhibitor), Dexamethasone (glucocorticoid)] for relapsed multiple myeloma, Cyclophosphamide, Adriamycin and fluorouracil (CAF) for metastatic breast cancer or Bleomycin, etoposide phosphate, cisplatin (BEP) for ovarian malignant cancer, … Several principles must be considered while setting up a strategy for combination therapies^[10]^. There must be no overlapping toxicities of the drugs used, their mechanisms of action must be clearly different, and there must be a minimal cross resistance. Drug solubility and permeation should also be similar in order to allow a good delivery and high intracellular levels. Eventually, the dose/ratios should be optimized in order to reach a synergetic effect between the different drugs. These last two conditions make very complex the design of combination therapies with different drugs. Furthermore, even if the overlapping toxicities are minimum, administration of several drugs at the same time (“cocktail administration”) induces high toxicity to normal tissue. To avoid that, the drugs can be administered sequentially which is not optimum for the patient^[11]^. The combination of different drugs within one nanocarrier might help solving these issues.

**Figure 1:**
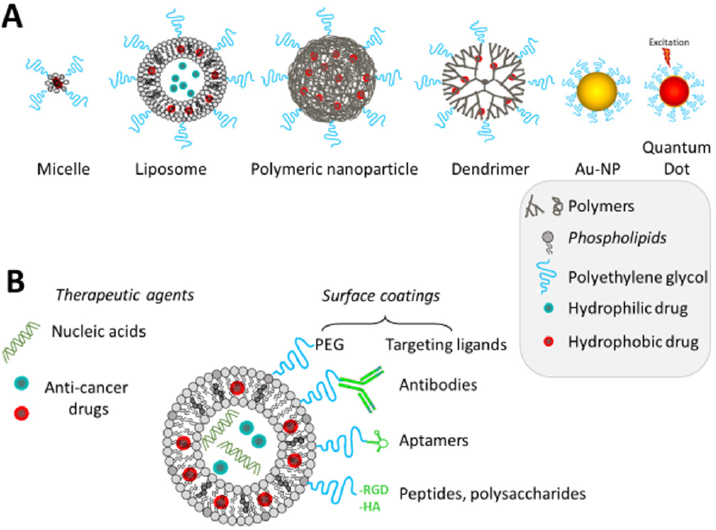
Main types and composition of nanocarriers used for cancer treatment and/or imaging (A); how can the nanocarrier be modified to target cancer cells (B)

Indeed, in the past few years, nanoparticles have become an interesting platform for medicine and treatment of cancers. They have several properties interesting for diagnosis and therapy [Figure 1]. First, they are multifunctional, so they can combine both diagnosis and therapy and though be used for personalized treatments or theranostic. Furthermore, they can have various structures, shapes and sizes. By modulating these parameters, an enhanced permeability and retention (EPR) effect can be observed: nanoparticles naturally accumulate into tumors due to the fenestrated neoendothelium and the low lymph drain in tumor tissue^[12]^. However, this effect is limited by the poor tumoral blood stream, the tumor type, the high tumor interstitial fluid pressure and the heterogeneity of the vascularization. Nevertheless, there are several methods to improve nanoparticles uptake by tumoral cells, and the efficacy against resistant cancers. Nanoparticles allow the protection of fragile molecules and help to have a controlled released in the target tissue.

### Improving nanoparticles tumor accumulation and selectivity by active targeting

One of the strategies to improve nanoparticles uptake by cells includes the use of active targeting. It involves a ligand specific of a cell-surface receptor linked to the nanoparticle^[13]^. This ligand can be a protein, a peptide, an aptamer, a natural binder or a small molecule. Lots of biomarkers has been studied and are interesting to target, like the markers of angiogenesis (integrins, NRPs, …)^[14]^, or some proteins expressed on the surface of cancerous cells (BCMA^[15]^, Muc1^[16]^, …). Recently, in numerous resistant cancers including pancreatic, breast, ovarian, prostate, colorectal cancers and neuroblastomas; CD44 has been identified as an interesting marker of cancer stem cell like cells. Yet, CD44 is involved in tumor progression, metastasis, drug resistance and its expression has been correlated to disease prognosis^[17]^. So researchers have been interested by targeting it, and developed different strategies to do so^[18]^. One of them is to use hyaluronic acid (HA), a natural agonist of CD44. It is a critical component of the extracellular matrix and it is degraded through enzymatic reactions^[19]^. So, it is bio-compatible. It has been used for surface modification of a nanoparticle, for instance Sun *et al.*^[20]^ designed a positively charged liposome, encapsulating a combination therapy composed by synergistic amount of NF-κB and STAT3 inhibitors (curcumin, celecoxib and a dual peptide TAT-NBD) for the therapy of metastatic inflammatory breast cancer. Thanks to the positive charges at the surface of the liposome, they coated it with HA which is negatively charged. They showed that the drug release was enhanced by hyaluronidase (HAase) and that the uptake was selective toward CD-44 expressing cells (*in vitro* and *in vivo*). Our lab developed several types of lipid based nanocarriers to target CD44-positive tumor cells using HA as a ligand and to deliver anticancer drugs or siRNA (for original research and reviews see^[21-24]^).

HA can also be used for self-assembled nanoparticles. Indeed, the conjugation of hydrophobic, or positively charged, moieties with hydrophilic, or negatively charged respectively, parts results in self-assembled nanoparticles^[20]^. For example Lee and Cho has grafted a hydrophobic analog of vitamin E (D-α-tocophenol succinate) to HA in order to give self-assembled nanostructures^[25]^. Moreover, they also grafted triphenylphosphonium moiety (cationic and lipophilic) to HA in order to add a mitochondrial targeting. In this nanostructure, they encapsulated lapatinib, a dual tyrosine kinase inhibitor of EGFR and HER2^[26]^. The targeting capacity of this system was demonstrated in an *in vivo* model of triple negative breast cancer. However, there are a receptor for endocytosis present in lung and liver which are responsible for early clearance of HA^[18]^. Chondroitin sulfate is another natural compound that has been reported to have a specificity for CD44 receptors. Thus, Zhang *et al.*^[27]^ used it to synthesized nanogels with a good affinity for CD44 receptors. They prepared a paclitaxel-chondroitin sulfate conjugates cross-linked with a glutathione (GSH) sensitive disulfide bridge to form the nanogel and loaded free paclitaxel and free sunitinib in synergistic ratios. Thanks to the GSH contained in cells the chondroitine sulfate conjugate will be degraded to allow release of drugs. First, they showed that the more tumor cells expressed CD44 receptors, the more the nanogel was internalized. Finally, they show a first burst release of the free drugs, once the nanogel internalized, and a sustained paclitaxel released from the conjugated drug. This system was set up to overcome drug efflux pomp resistance. The use of antibodies is also an excellent approach for targeted delivery. To target CD44, Lu *et al.*^[28]^ conjugated an anti CD44 antibody to a liposome through a thiol-maleimide reaction. They encapsulated a steroin saponin TAIII, a hydrophobic antitumoral drug candidate. They demonstrated that this encapsulation improved the solubility and the bioavailability of the drug. Furthermore, they showed the tumor accumulation thanks to the CD44 targeting: an improved *in vitro* uptake of HepG2 cells was observed by fluorescence of the targeted liposome compare to the unconjugated one. They also showed a greater accumulation of these liposomes in tumoral site in HepG2 xenograft mice. However, an antibody is a large protein with only a small specific fragment. Another strategy is to conjugate only the specific fragment in order not to change the behavior of the nanoparticles. Another group chose to re-engineer the Fab fragment of a human anti-CD44v6 antibody by adding 3 more cysteine. This allow them to conjugate it with a maleimide-nanoparticle to have a better binding affinity^[29]^. The fragment they used had an affinity of 2-6 nmol/L for CD44v6 and 6-70 nmol/L for the other proteins of the family. After re-engineering, the affinity was of 10-90 nmol/L, then they validated the affinity after conjugation with the nanoparticle. With the same type of conjugation (thiol-maleimide) the targeting of CD44 has been done in our group using an aptamer^[30,31]^. The free anti-CD44 RNA-based aptamer has a binding affinity around 21 nmol/L, whereas the liposome conjugated to the aptamer has an affinity of 6 nmol/L^[32,33]^. This can be explained by multivalent binding of the liposomes to CD44. Another system used a layer of chitosan, a polysaccharide biocompatible and biodegradable, to target CD44^[34]^. They developed a multilayer system, allowing to deliver 3 drugs in the same time (doxorubicin, paclitaxel and silybin) for the treatment of drug-resistant breast cancer.

### Nanoparticles to improve the delivery for combinations of therapies

Nanocarriers are useful for drug delivery system, and therefore for combination of drugs. First, they can overcome drug-solubility problems by conjugation or encapsulation of hydrophobic drugs (liposomes, polymers, …), therefore they improve the internalization of the drug itself by several mechanism of internalization; secondly, the conjugation of the drug can inactivate the drug into a prodrug, and thus decrease the systemic toxicity. In that case, the drug can be released by a specific trigger once in the microenvironment of the tumor (pH, redox, heat, IR…) or in the cell. These release strategies are detailed in a recent review by Zhou *et al.*^[35]^. Finally, in the case of combination therapies, it allows the delivery at the same site of all the drugs, and also at a determined synergistic ratio^[36]^.

Furthermore, they allow the combination of several types of therapy^[8]^. Indeed, like nanoplatforms are multimodal, they can combine several properties. For example, the delivery of gene therapy is challenging because naked RNA and DNA are fast degraded in plasma. However, nanoparticles can protect them until delivery, and combine them with other therapies. It also can be used for photodynamic therapy, either by properties of the nanoparticle itself (nanoporphyrin) or by the surface functionalization with a photosensitizer. On the same idea, iron oxide nanoparticles (ION) can be used for photothermal therapy, and other type of nanoparticles can be used for immunotherapy (drug-conjugate…).

In that line of idea, Chen *et al.*^[37]^ developed an ultrasound triggered multimodal nanoparticle. They designed it for metastatic colorectal cancer: they formed microbubbles (used as contrast agent for ultrasound imaging) by combining porphyrin grafted lipid (enables photodynamic therapy and fluorescent imaging) and amphiphilic drug conjugate (hydrophobic camptothecin conjugated with hydrophilic fluouridine by a hydrolysable ester function). This technique allowed really stable nanoparticles (no early release) with a great drug loading content. Once the microbubbles, followed by ultrasound imaging, are at the tumor site, they are converted into nanoparticles by ultrasound exposure. Thus, they are internalized into the cells, which the acidic environment enables the hydrolysis of the ester function and thus, the dissociation of the drug-drug conjugate. Furthermore, the use of the ultrasound combined to the PDT reduces the expression of ABCG2, a drug pomp efflux responsible for MDR.

The conversion of nanoparticles by an external stimulus is also a strategy used to overcome barriers of resistant cancer.

### Nanoparticles to overcome barriers of resistant cancer

Nanoparticles can also improve drug-delivery to resistant tumors by overcoming pathophysiological barriers. This is well explained in a recent review by Liu *et al.*^[9]^. Nanoparticles can overcome extracellular barriers by adapting their size, their surface, or the drug leakage/prodrug according to different triggers in the tumor microenvironment: more acidic pH (6.5-7.2), an increase of redox potentials and MMPs overactivated. Some nanoplatforms can also target the tumor micro-environment: either by degradation of the extracellular matrix, either by a phototherapy-induce alteration of the microenvironment. Finally, they can overcome intracellular barriers, by getting free of lysosomes by different pathways (light excitation, …).

### Bio-mimetic nanoparticles to overcome drug resistance in cancer

Although nanoparticles are promising platforms for therapy, most of them are cleared prematurely from the system, by opsonization, and uptaken by the macrophages^[38]^. Thus, it is important to design stealthy nanoparticles. To palliate to this problem, some researchers focused on biology to develop bio-inspired nanoparticles which then are bio-compatible and stable in plasma and are naturally targeting some type of cells. They thought about camouflage nanoparticles with cell membranes. Though, Mu *et al.*^[39]^ used stem cell membranes to coat magnetic nanoparticles of iron oxide. Therefore, they obtained an image guided, photothermal, siRNA delivery platform. They validated the presence of membrane proteins on their coated nanoparticles by SDS-PAGE, and the capability of the platform to induce a thermal increase after activation by NIR and to deliver siRNA. The increase in cellular uptake was shown by fluorescence microscopy and flow cytometry *in vitro*. *In vivo*, they showed the ability of the system to image the tumor by MRI 24 h after the injection. The biodistribution have been improved compare to the iron oxide alone: although the accumulation into heart; lung and kidney was the same, the coating decreased liver and spleen accumulation and increased the tumor uptake significantly. With a similar strategy, Cheng *et al.*^[40]^ used a circulating tumor cells’ membrane to coat metal-organic framework nanoparticle. This system allows encapsulation of therapeutic proteins, here gelonin. They optimized the synthesis to obtain 100-nm nanoparticles and showed an *in vitro* decrease of the uptake of nanoparticles by macrophages, and an increase uptake by tumor cells. The selectivity towards tumor cells was proved. Indeed, healthy murine cells didn’t show any uptake *in vitro*. The *in vivo* biodistribution showed an increased tumor uptake and a decreased uptake by liver, and a better treatment efficiency than MOF or proteins treatment. Finally, He *et al.*^[41]^ combined membranes from two cell types (tumor cells and leukocyte) in order to combine the ability to thwart the detection by immune system of the leukocytes and the homotypic targeting of the tumor cells. They confirmed the combination of the two membranes by fluorescence and the presence of the cell biomarkers. They encapsulate Paclitaxel into these leutusomes. They show that the leukocyte membranes decreased the leukocyte uptake of the nanoparticles and that the tumor cell membrane increased the tumor cells uptake *in vitro*. They show a decreased in tumor size *in vivo* and no systemic toxicity.

## Nanoparticles into clinics: what are the limits?

Although nanoparticles have been wild investigated this last few years for therapeutic applications, few of them have been FDA approved. Indeed, the lack of established standard for the evaluation of nanoparticles limits their developments into clinics. During a workshop, researchers tried to define the challenges and some solutions and report it into a review^[42]^.

The challenges of nanomedicine start as soon as the design of the treatment: indeed, nanoparticles solution is a distribution of size, shape, molecular weight, … Therefore, it complicates their physico-chemical characterization, and the establishment of their properties. It raises the question of what the criteria are to consider two different synthesis batches of nanoparticles as the same. Mülhopt *et al.*^[43]^ realized studies on a large scale of nanomaterial of the variability of a large numbers of parameters. Although size is generally well controlled, and measurements repeatable, other parameters, like impurities are more variable and impacts on Reactive Oxygen Species (ROS) generation for example.

Furthermore, despite the EPR effect, a small amount of the injected dose really reached the tumor site. Wilhelm *et al.*^[44]^ showed on a large data set that regardless of the nanoparticle size, the delivery efficiency to the tumor site do not exceed 1%. This is not a problem if the nanoparticles injected aren’t toxic, but it is a challenge for drug delivery. Indeed, it has been shown that nanoparticles have a premature clearance, by several mechanisms. Blanco *et al.*^[38]^ have studied the biodistribution of nanoparticles according to “4S parameters”: shape, size, stiffness and surface functionalization. Spherical nanoparticles under 5 nm are cleared by renal elimination. The other ones tend to accumulate into liver or lung. Most nanoparticles are opsonized and sequestered by the mononuclear phagocyte system. The challenge is thus to extend the blood circulation of nanoparticles, and to define the toxicity of nanoparticles for the organ into which they accumulate. Indeed, the toxicity of nanomaterials is still not well known; particularly for the liver, though, the toxicity toward hepatic cells should be well studied^[45]^.

When the nanosystem has been proved to be relevant in pre-clinical studies, there is still lots of barriers for the translational development: the scale-up is often difficult, regulatory and money, safety and efficacy, clinical adoption^[46]^.

## Inhibition of HSP90 isoforms, a promising approach to potentiate conventional therapy

### HSP90 family

HSPs are a family of proteins, classified by their weight, discovered in the laboratory of Ferrucio Ritossa^[47]^. The HSP90 family are highly conserved and localized in different organelles (inducible HSP90-α and constitutive HSP90-β in the cytosol, GRP94 in the endoplasmic reticulum, and TRAP1 in the mitochondria).

These proteins are part of a chaperone machinery^[48]^, achieving stabilization and maturation of client proteins (over 200 for HSP90α/β, actualized list of the cytosolic HSP90 clients available at https://www.picard.ch/downloads/HSP90interactors.pdf). This is possible by a cycle involving co-chaperones and other heat-shocks proteins. Thus, a complex formed by HSP70 and HSP 40 allows to partially fold some client proteins, and to transfer it to HSP90 thanks to a co-chaperone: HOP. Others co-chaperones are involved during the cycles: they bind to a specific conformation of HSP90, in a sequential manner. They stabilize HSP90 in a given conformation, regulates ATPase activity of HSP90, though regulates the cycle of HSP90… Depending on the type of client protein, the co-chaperone implicated in the machinery can be different. The HSP90 proteins have mainly 3 domains: the N-terminal domain which is responsible for ATP-binding, the middle domain which allows the binding of co-chaperone and client proteins and the C-terminal domain which enables the dimerization of HSP90 proteins.

The expression of HSP90 and other heat-shocks proteins are regulated by a transcriptional factor: HSF-1. But HSF-1 is also a client protein of HSP70 and HSP90. The complex formed by these 3 proteins keeps HSF-1 in an inactive state. In case of stress, HSF-1 is released and enables the Heat Shock Response (HSR) to induce HSP genes. Other regulations involving post-translational modifications are explained in a review by Prodromou^[49]^.

**Figure 2:**
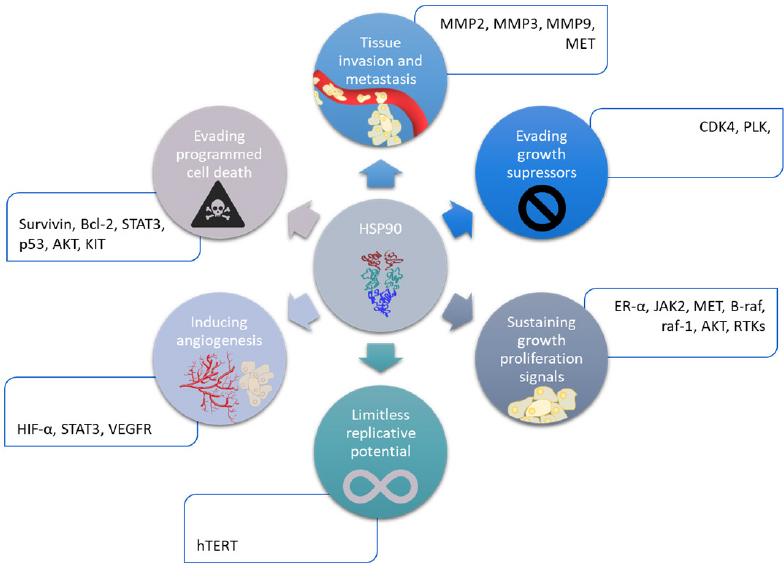
Implication of HSP90 family in the hallmarks of cancer. HIF-α: hypoxia inducible factor α

HSP90 has various client proteins, involved in several important pathways like apoptosis, cell cycle control, cell viability and signaling events. These client proteins are summarized in Figure 2. Furthermore, their overexpression has been linked to several types of cancer (breast, melanoma, leukemia, colon carcinoma, non-small-cell lung cancer, prostate cancer…)^[48]^. Thus, this family is an interesting target to overcome resistance. Indeed, like they are involved in all the hallmarks of cancer, and several pathways of intracellular signaling. Among them, there are survival and proliferation pathways, detailed above. The client proteins of HSP90 involved in drug resistance are listed below, in Table 1. Furthermore, the HSP90 chaperone machinery is able to avoid misfolding and degradation of mutated and overexpressed proteins, hence promoting cancer cell survival^[50]^.

**Table 1:** Summary of different HSP90 client proteins involved in drug resistance mechanisms^[48,50,51,52]^

Class of proteins or pathway		Client protein of HSP90 family	Involved in mechanism resistance	Implicated cancers
Surface receptors		HER2	Promotes cell proliferation and opposes apoptosis	Breast, ovarian
		EGFR	Promotes cell migration, adhesion and proliferation	NCSLC and glioblastoma
		VEGFR	Promotes angiogenesis and cell migration	Various cancers
		KIT	Activation of pro-survival pathway	GIST
Cell signaling	PI3K/AKT/mTOR	AKT	Intracellular signaling involved in the regulation of apoptosis, cellular cycle and angiogenesis	Lung
		FLT-3		AML
		MET		Melanoma, gastric, lung
		JAK2		Lymphoma
	RAF/MEK/ERK	Raf1	Signaling proliferation	Melanoma
		B-raf		Melanoma
		MET		Melanoma
		JAK2		Lymphoma
Transcription factors		HIF-α	Promoting angiogenesis	Renal
		ER-α	Regulating genes involved in cellular proliferation	Breast
		p53	Transcription of genes involved in cell cycle arrest or apoptosis	50% of cancers
		STAT3	Apoptosis, immunity and angiogenesis	
Damage response		BRCA1/BRCA2	Transcription regulation, DNA repair and ubiquitination	Breast, ovarian, prostate
		RAD51	DNA repair	
		Chk1	DNA damage response and cycle checkpoint response	AML
Apoptosis		Bcl-2	Regulates mitochondrial apoptotic pathway	Follicular lymphoma/SCLC
		Survivin	Inhibitor of apoptosis	GBM
Extracellular matrix		MMP2, MMP3, MMP9	Facilitates invasion through cell adhesion, matrix digestion and cell migration	Various cancers
		FAK	Actin-based cell motility	Breast, colon
Kinases		PLK	Ser/Thr protein kinase - Cell regulation (G2/M trigger)	Lung, colon, AML
		CDK4	Cytokine kinase - cell cycle regulation	CML
Chimeric fusion protein		BCR-ABL	Activates numerous signal transduction pathways in leukemogenesis	CML
		NPM-ALK	Induces cell transformation and proliferation	Anaplastic lymphoma

NCSLC: non small cell lung cancer; GIST: gastrointestinal stromal tumor; AML: acute myeloid leukemia; GBM: glioblastoma; CML: chronic myeloid leukemia

In the last few years, HSP90 inhibitors were developed, with no paralog specificity but a binding capacity to the N-terminal domain of the protein. Several inhibitors went to clinical trials (over 50 clinical trials with 15 different N-terminal inhibitors^[53,54]^), but none were FDA approved. Indeed, the N-terminal binding induces a HSR by releasing HSF1 of HSP90, and thus a resistance to HSP90 inhibition^[55]^. Hence, several strategies emerged; the targeting of HSP90 by the C-terminal domain, or the middle domain; and the specific inhibition of a paralog. Indeed, Wang and McAlpine showed that the domain targeted have an influence on the phenotype and the heat-shock response^[53]^. They compared the effect of two chemical inhibitors on HCT-116 cells: 17-AAG (GI_50_ = 50 nmol/L), which binds to the N-terminal domain; and SM122 (GI_50_ = 8 µmol/L) which binds to the C-terminal domain. They compared the activity of these two inhibitors and a heat shock event at 3 levels: the accumulation of unfolded proteins, the expression of 3 mRNA, and the transcription of 3 proteins. Interestingly, the level of unfolded proteins increased significantly whereas the cells were treated with 17-AAG or SM122, and the result of these treatments were similar to a heat shock event. Then, they focused on the mRNA expression of inducible HSP70 (gene HSPA1A), constitutively expressed HSC70 (HSPPA8) and HSP27 (HSB1). Both treatments with 17-AAG and heat-shock event gave similar results, with an increased expression of these 3 genes whereas the treatment with SM122 showed a down-regulation of these three genes. Finally, the treatment with 17-AAG and the heat shock event both induced an increased level of HSP70, HSP27 and HSF1 proteins, although the treatment with SM122 showed a decrease protein level for the same ones. To conclude, N-terminal and C-terminal modulators have different phenotype.

Afterwards, some groups focused on C-terminal binding inhibitors, and showed its activity without HSR. Alami/Messaoudi^[56-59]^ and Blagg^[60,61]^ groups developed independently some C-terminal inhibitors based on a natural inhibitor: novobiocin. Terracciano *et al.*^[62]^ developed two other inhibitors, different from novobiocin, and shows that they induces different kinds of response than novobiocin based inhibitors but no HSR as well. At last, some groups studied the inhibition by the middle domain and found no HSR and an induction of apoptosis^[63]^. Bhatia *et al.*^[64]^ showed the interest of C-terminal inhibitor to tackle imatinib-resistant chronic myeloid leukemia (CML). Hyun *et al.*^[65]^ designed a new C-terminal inhibitor by mixing structures of two inhibitors of HSP90 in order to circumvent drawbacks of both inhibitors: novobiocin, which has a high IC_50_ of 700 µmol/L, and deguelin, which has important sides toxic effect. This new inhibitor showed inhibitory effects on viability and colony formation on NSCLC cells and chemoresistant cells, whereas it showed minimal effect on viability of normal cells.

This kind of inhibitors are exhaustively described in a review^[66]^, and they have shown to sensitize cancer cells to lots of chemotherapies (in various cancer: breast, prostate, ovarian, melanoma, glioblastoma, leukemia, myeloma,…)^[67]^.

Another strategy to improve HSP90 inhibitors efficacy in translational medicine is to selectively target isoforms, either by improving the specificity towards the targets (crystal studies, …), either by targeting an organelle (intracellular compartment). These more selective inhibitors would have more specific actions, and thus would be less toxic.

## Inhibition of organelle specific isoforms

### HSP90 alpha/beta

To decrease the HSP90-inhibition related toxicological effects, the selective inhibition of HSP90α/β allows to reduce the number of client proteins involved, and not to touch to the functions of the mitochondria and the endothelial reticulum. So, Ernst *et al.*^[68]^ developed a specific inhibitor by studying the conformations of the different isoforms. The inhibitor developed shows a binding affinity of 5 nmol/L with HSP90α/β, whereas those of GRP94 and TRAP1 are above 10 µmol/L. This strategy is efficient for some diseases (like Huntington’s one), but its efficacy has not been proved yet in oncology and resistant cancers. Most of the groups have focused on the specific targeting of the other organelles (ER and mitochondria), which have less client proteins and so their inhibitors could be less toxic for healthy tissues. Furthermore, although the expression of HSP90-α is not critical for healthy cell viability, the one of HSP90-β is^[69]^. Whereas, the expression of both GRP94 and TRAP1 aren’t vital for healthy cells^[70,71]^.

### GRP94

GRP94 is mainly located in the ER and shares 50% homology with HSP90 cytosolic^[47]^. It has the same ATPase activity as cytosolic HSP90, however, it is the major calcium binding protein in the ER and has specific limited client proteins. GRP94 is involved in calcium homeostasis and ER quality control (protein folding, interaction with the protein folding machinery, calcium storage…).

GRP94 is involved in several pathways responsible for some drug resistances^[72]^. Indeed, it maintains ER Ca^2+^ homeostasis and thus protects cancer cells from apoptosis. It’s also involved in PI3K-AKT pathway by binding some insulin-like growth factor (IGF1, IGF2) to their receptors. Furthermore, some integrins are also client proteins of GRP94, and are linked to invasion and metastasis. All these pathways can explain why GRP94 is linked to drug resistance in lots of tumors.

Blagg group has worked on specific GRP94 selective inhibitions for a few years. They began to crystallize GRP94 and HSP90 to identify differences^[73]^. From that they determine 3 interesting scaffolds for this targeting (radamide, purine, NECA). Thus, they developed first GRP94 inhibitors from the radamide scaffold, with a binding affinity around 820 nmol/L for GRP94 and a good selectivity toward HSP90α^[74]^. In the same time, another group worked on GRP94 inhibitors from purine-scaffold, and prove its selectivity with a binding affinity of 220 nmol/L for GRP94, 7 µmol/L for TRAP1 and above 25 µmol/L for HSP90α/β^[75]^. Once the first inhibitors developed, they focus on NECA scaffold and the interaction with other structures to increase the binding efficiency, and create a new inhibitor with a 200-fold selectivity (binding affinity of 440 nmol/L for GRP94 and above 100 µmol/L for HSP90α)^[76]^. Afterwards, with the interaction studies of these inhibitors, they discovered new hydrophobic subpocket of GRP94, which allows a new type of GRP94 inhibitors scaffold: the resorcinol one. The first one showing a binding activity of 8 nmol/L with GRP94, and of 77 nmol/L with HSP90α^[77]^. The second one showing a binding affinity of 4 µmol/L with GRP94, and no affinity for HSP90α for the range of concentration tested. They showed *in vitro* with another resorcinol inhibitor (binding affinity of 0.54 µmol/L with GRP94 and a 73-fold selectivity towards other HSP90s) that the selective inhibition of GRP94 could decrease migratory abilities of cancer cells and thus be really useful in aggressive cancers^[78]^. Finally, they showed *in vivo*, with an open-angle glaucoma model that the selective inhibition of GRP94 shows less toxicities and undesirable effects^[79]^.

### TRAP1

TRAP1 shares high homology with cytosolic HSP90, reflecting 34% identity and 60% similarity. However, it binds ATP with 10-fold affinity than HSP90^[47]^. Structurally, TRAP1 doesn’t have a C-terminal MEEVD motif and a charger linker domain connecting Middle Domain to C-terminal Domain, both found in HSP90α/β. Furthermore, TRAP1 has a major role in the mitochondria: it maintains its integrity and protect against mitochondrial apoptosis^[80-83]^. It also protects against cell death by overproduction of ROS and inhibits oxidative phosphorylation by interacting with complexes of the respiratory chain (Succinate DeHydrogenase and cytochrome oxidase). These two last functions are involved in drug resistance mechanisms. TRAP 1 has been shown to be responsible for resistance to apoptosis and involved in MDR in several cancers (colorectal cancer^[80,84]^, thyroid carcinoma^[81]^, breast cancer, NSCLC^[82]^, ovarian cancer, prostatic cancer, …). So, the selective inhibition of TRAP1 can be interesting for resistant and aggressive cancers. Like GRP94, some studies on the crystal structures of TRAP1 interactions (mainly focused on the N-terminal domain of the protein), allowed researchers to develop a specific inhibitor to TRAP1^[85]^. They changed the purine ring of an HSP90 inhibitor into a pyrazolopyrimidine in order to decrease the binding of this inhibitor with HSP90 cytosolic and so increased its concentration into the mitochondria: thus, its binding affinity with TRAP1 is of 79 nmol/L, whereas it is of 698 nmol/L for other analogs. Other groups developed specific TRAP1 inhibitors by using a mitochondrial delivery vehicle. Thus, in 2005 the Altieri group demonstrated that Shepherdin, a 17-AAG derivative carrying the antennapedia peptide, efficiently accumulates inside the mitochondria and inhibits the chaperone activity of TRAP-1 and HSP90 through a competition with ATP thus resulting in cell death involving the regulation of the mitochondrial transition pore opening^[86]^.

More recently, the same group developed gamitrinibs^[87]^. They combine an HSP90 inhibitor (17-AAG) with various linkers’ length and a mitochondrial targeting moieties triphenylphosphium (TPP) as well as a guanidinium salts. The length of the linker (alkyle chains most of the time) influences on the mitochondrial matrix accumulation of the drug^[88]^, a long chain increases the octanol/water partition and thus the mitochondrial accumulation. The targeted 17-AAG derivated inhibitor has an IC_50_ between 4 and 20 µmol/L depending of cell-lines since the 17-AAG as a non-targeted drug has an IC-50 between 25 and over 200 µmol/L for the same cell lines confirming the rationale to target mitochondria to enhance activity of HSP90 inhibitors. Another group combined both approaches, hence combining a specific inhibitor of TRAP1 to a mitochondrial delivery vehicle^[89]^. They started with a 3,4 isoxazole diamide structure, to which they added spacers of various length and a cationic head (triphenylphosphonium, pyridinium salts, guanidinium, polyamine, …). For now, they have similar binding affinities (in the nmol/L range) toward HSP90 and TRAP1, and thus need to be improved thanks to the crystal structure of TRAP1 (full length). However, they show an accumulation into the mitochondria, and good ATPase inhibition (IC_50_ around 500 nmol/L), an important cell proliferation inhibition (IC_50_ = 1.32 µmol/L) and an induction of apoptosis starting as soon as 1 µmol/L.

The conjugate Gamitrinib - triphenylphosphonium head has been studied *in vitro* and *in vivo*. It has been shown that it induces apoptosis, cell cycle arrest in G2-M phase, and an increase in ROS levels in liver cancer^[90]^. It has also been shown that it induces death in hepatocellular carcinoma cells through a combination of death pathways: necroptosis, apoptosis and autophagy^[91]^. Furthermore, G-TPP has shown good synergistic properties with Bcl-2 inhibitors^[92]^. As well, preclinical studies have been done in prostate cancer, and G-TPP has shown a good inhibition of cell growth (GI_50_ between 1.10^-7^ and 5.10^-5^ mol/L) and a loss of metabolic activity with prostate cancer cells, multi-drug resistance prostate cancer cells and bone metastatic prostate cancer cells. This has also been tested *in vivo*^[93]^. No significant systemic or organ toxicity was observed.

## Why targeting TRAP1 protein to fight cancer?

The intrinsic signal pathway for cell apoptosis and energetic metabolism (respiration and Krebs cycle) are mediated by mitochondria. Furthermore, TRAP1 takes part of a pro-survival cell pathway, which may lead to therapeutic resistance. Thus, it’s an interesting organelle for targeted therapeutics^[94]^. It has been shown that TRAP1 is involved in resistance to DNA damaged induced by cis-platin and H_2_O_2_^[80]^. Up to now, TRAP-1 has been mainly considered as an oncogenic protein in many cancers (prostate, colorectal and breast cancers) but recently some evidences are in favor of the tumor suppressor role of TRAP-1 in some cancers as in ovarian cancer. That’s why inhibition of TRAP-1 has to be chosen taking into account the potential role in tumor growth or survival^[95]^.

TRAP1 inhibits the succinate dehydrogenase, which stabilizes the factor Hypoxia Inducible Factor 1α (HIF 1α) and though allow the neoplastic^[83,96]^. It also inhibits CYPD, so the permeability transition pore (PTP) can’t open anymore, and the death signal isn’t able to go into the cytosol anymore. Moreover, it inhibits the production of ROS species by binding with two complexes of the mitochondrial respiration (II and IV).

The Warburg effect, considered as a marker of cancerous cells for a long time, is not a universal feature of cancer anymore. Some tumors preferentially rely on oxidative phosphorylation mechanism rather than the aerobic glycolysis, preferred usually by cancer cells. Interestingly, some researches show that whereas TRAP1 favors oncogenic phenotypes in glycolytic tumors, it was down-regulated in tumors relying on oxidative mechanisms^[97]^. This correlates with the fact that TRAP1 has been shown by Yoshida *et al.*^[70]^ to regulate the cellular respiration: a reduced or absent expression of TRAP1 would favorize oxidative phosphorylation whereas a high expression of TRAP1 would favorize aerobic glycolysis^[70]^.

Some studies showed the presence of TRAP1 in the ER, and enlighten its involvement in ER stress protection and therefore the interest of targeting endoplasmic TRAP1 to overcome drug resistance^[98]^.

Although all the inhibitors described above and targeting HSP90 isoforms are promising, most of them are water insoluble. Thus, administration of those drugs is limited. A solution to this problem is to use nanoparticles to administer these drugs, Furthermore, this could allow to deliver a combination therapy with more precision, and to actively and selectively target tumors in order to avoid systemic and organ toxicity.

## Will a combination of both HSP90 inhibition and the use of drug delivery nanosystems give advantages to treat aggressive and drug resistant cancer?

### Inhibitors of HSP90 in combination for drug resistance

HSP90 has been tested in combination therapies by several groups and has shown great synergism with different other drugs, to overcome resistances. These studies are summarized in Table 2. The combination of both is thus relevant to overcome resistances.

**Table 2:** Impact of HSP90 inhibitors on client protein-based resistance pathways

HSP90 inhibitor	Type of cancer/clinical phase	Combinations with	Overcoming resistance to	Client proteins/pathway involved	Ref.
17-AAG (Benzoquinone derived)	Human melanoma/ Preclinical	PI3K/mTORinhibitors	BRAF inhibitors mTOR inhibitors	PI3K/AKT/mTOR and RAS/RAF/MAPK	[99]
3-COA (Oleaonolic acid)	Lung cancer (Preclinical)	Cisplatin or Doxorubicin	Cks1b-induced chemoresistance (Cisplatin and doxorubicine)	AKT/MEK	[100]
AUY922 (resorcinol derived)	P53-mutant Head and neck cancer (preclinical)	Concurrent Cisplatin radiotherapy	DNA damages	p53, RAD51, ChK1, BRCA1	[101]
	Mantle cell lymphoma (Preclinical/phase1/phase2)	Preclinical, alone	Ibrutinib (bruton tyrosine kinase inhibitor)	Bruton tyrosine kinase, JAK2	[102]
	KRAS-mutant NSCLC (preclinical)	GSK458 (PI3K inhibitor)	PI3K inhibitor	AKT and RAF	[103]
	KRAS-mutant NSCLC (Preclinical)	Trametinib (MEK inhibitor)	MEK inhibitors	AKT and RAF	[104]
	Breast cancer (Preclinical)	Fulvestrant	Hormone treatments	ErbB receptors	[105]
AUY922/AT13387 (HSP90/ALK inhibitor)	Lung adenocarcinoma (*In vitro* studies)	Dual targeting inhibitor	ALK inhibitors	HER2, ATK, ALK	[106]
Gamitrinib (TRAP1 inhibitor)			Apoptosis resistance	Bcl-2	[91]
Ganetespib	Breast cancer (preclinical studies)	ABT-888 (PARP inhibitor) and ionizing radiation	PARP inhibitors	Core proteins in the DNA repair machinery (BRCA1, BRCA2, RAD51)	[107]
	Breast cancer (preclinical studies)	Zinc phtalocyanine conjugate (photosensitizer)	ROS	HSP90 extracellular (targeting) HIF 1α, surviving, VEGF	[108]
	Metastatic breast cancer (phase I clinical trials)	Paclitaxel and trastuzumab (anti-HER2 antibody)	Anti-HER2 resistance	RTKs	[109]
KW-2478	Multiple Myeloma (Phase I/II study)	Bortezomib	Relapsed/refractory		[110]

### HSP90 targeting-nanoparticles to sensitize cancerous cells

Some HSP90 targeting-nanoparticles have already been developed, and detailed in a previous review by Sauvage *et al.*^[111]^. Since this review, the nanoparticles have been improved, and new ones had been designed. There is still few of them designed with an organelle specific inhibitor, but pan-inhibitors have been incorporated into nanoparticles to sensitize resistant cancers.

The pan-inhibitor 17-AAG has been encapsulated in micelles by Le *et al.*^[112]^. It was co-encapsulated with rapamycin, a mTOR inhibitor, and docetaxel, an anti-mitotic. They designed this nanoparticle to target multiple pathways in order to treat advanced prostate cancers which are resistant to all current therapies. They have shown that this system targets simultaneously several signaling axes, including mTOR and PIK3/AKT pathways. This led to increased cytotoxicity effects and to an *in vitro* and *in vivo* growth tumor inhibition by caspase-dependent pathway. 17-AAG has also been integrated to a nanogel with another combination therapy as reported by Shin *et al.*^[113]^: Epothilone B, an inhibitor of microtubules, and rapamycin. This formulation has been developed to overcome chemoresistance against microtubules stabilizing agents in an ovarian tumor model. They found an optimistic ratio of these three drugs (0.4:3.0:1.5, EpoB/17AAG/Rap), which exhibits a strong synergy with an IC_50_ of 3 nmol/L, and a decreased clonogenicity. This formulation is thermosensitive, which allows a triggered release of the drugs, and so less systemic and organ toxicity. *In vivo*, it has been safely injected by i.p., and a decreased of metastasis has been observed.

However, nanosystems not only enable easy combination therapy delivery, but also enable combination of several types of therapies. Thus, Peng *et al.*^[114]^ developed a thermoresponsive polymer to achieve both chemotherapy (17-AAG) and photothermal therapy (IR780, photosensitizer) at the tumor site. Release of drugs is triggered by heat, while IR780 is excited for the photothermal ablation therapy (polymer micelles reaches 50 °C). This system has been designed for overcoming resistance to heat shock event. A synergistic cell death has been noticed *in vitro* in colon cancer cell lines, as well as a suppression of growth tumor *in vivo*.

Another system allows the combination of three types of therapy: photothermal therapy (PTT), photodynamic therapy (PDT) and chemotherapy. It is a nanoporphyrin platform loaded with 17-AAG^[115]^. HSP90 sensitizes cells to ROS species and heat shock event. This platform showed a strong synergistic inhibition of cell proliferation. The authors studied the molecular mechanism, and found an increase of HSP70, as well as a decrease of HIF 1α, ERK, AKT and Src decrease, characteristic of an HSP90 inhibition by a N-terminal modulator. Even if there was no active targeting, a monitoring by NIR imaging was possible thanks to porphyrins physical properties. This allowed an excitation of the porphyrin when accumulated into the tumor site.

Although the previous systems encapsulated a N-terminal inhibitor, we developed a liposomal system in which a C-terminal inhibitor 6-BrCaQ^[116,117]^ has been encapsulated as well. It has been encapsulated in liposomes and administered *in vivo* to mice bearing a triple negative breast cancer model. *In vitro* studies showed an induction of apoptosis, an anti-proliferation effect, a decrease of cellular migration and a decrease of the levels of client proteins of HSP90. *In vivo* studies showed a decrease of tumoral growth. Another group formulated pH-responsive polymeric nanoparticles with a new compound, a polar cyclic peptide (LB76) identified as a C-terminal inhibitor of HSP90 but unable to cross cell membranes. These particles allow endocytosis and favorize activity in a colorectal cancer cell line^[118]^.

To induce drug sensitivity to late stages resistant cancer, Banerjee group developed a glucocorticoid liposomal delivery system^[119,120]^ which target HSP90 by a mRNA. This lipoplex coformulated an anti-HSP90 mRNA (amiR-HSP90) and a drug which down-regulates mTOR pathway (ESC8). This liposomal formulation presented dexamethasone to target glucocorticoid receptor, overexpressed in cancer cells. This system has been tested in pancreas cancer cell lines and melanoma cancer cell lines and show antitumor activity. More interestingly, it shows a reverse drug resistance by downregulating a drug transporter protein: ABCG2. Thus, it showed impressive synergistic effect with doxorubicin.

## What’s next?

Whereas pan-HSP90 inhibitors have been encapsulated in various nanosystems, there are few nanoparticles for the delivery of organelle specific inhibitor. This strategy could be imagined either by encapsulating a specific inhibitor in a nanoplatform, either by adding an organelle targeting on the surface of the nanoparticle. For instance, some groups have worked on nanosystems presenting both CD44 targeting and mitochondrial targeting^[25,121-124]^. These dual targeting could allow to significantly reduce toxicity: the drug is delivered to the tumor site, which reduce systemic toxicity, and then the targeting of mitochondria could allow a specific inhibition of TRAP1.

## Conclusion

In this review, we highlighted the HSP90 chaperone machinery as an interesting target for drug resistant cancer, as well as the importance of nanocarriers to overcome physiological barriers to deliver therapeutics and to improve drug efficacy without increasing side effects. We underlined the usefulness of nanoparticles to administer combination therapies, to improve the efficiency of the treatment. Moreover, these systems can target a specific biomarker, which allow a specific delivery to cells responsible for the cancer invasiveness (stem cell-like cancer cells, metastatic cells…). We strongly believed that the future treatments should rely on the use of an organelle specific HSP90 inhibitor combine to nanotechnology to improved targeting, and eventually combination therapies.

## Declarations

### Copyright

© The Author(s) 2019.
